# Plasma myeloperoxidase: association with atrial fibrillation progression and recurrence after catheter ablation

**DOI:** 10.3389/fcvm.2023.1150324

**Published:** 2023-08-31

**Authors:** Jingmeng Liu, Changjian Lin, Taojie Zhou, Yangyang Bao, Yun Xie, Yue Wei, Qingzhi Luo, Tianyou Ling, Wenqi Pan, Ning Zhang, Lin Lu, Liqun Wu, Qi Jin

**Affiliations:** ^1^ Department of Cardiovascular Medicine, Ruijin Hospital, Shanghai Jiao Tong University School of Medicine, Shanghai, China; ^2^ Institute of Cardiovascular Diseases, Ruijin Hospital, Shanghai Jiao Tong University School of Medicine, Shanghai, China

**Keywords:** atrial fibrillation, myeloperoxidase, left atrial volume, catheter ablation, recurrence

## Abstract

**Background:**

Myeloperoxidase (MPO), released by activated neutrophils, is significantly increased in atrial fibrillation (AF). MPO may play a role in the progression of atrial fibrillation and further involved in AF recurrence after catheter ablation. We compared plasma MPO levels in paroxysmal and persistent AF and explored their role in AF recurrence after catheter ablation.

**Methods:**

Plasma MPO levels were measured in consecutive patients with paroxysmal AF (*n* = 225) and persistent AF (*n* = 106). Samples of patients were collected from the femoral vein during catheter ablation and all patients included were followed up after catheter ablation.

**Results:**

Plasma MPO levels increased from paroxysmal AF to persistent AF patients (56.31 [40.33–73.51] vs. 64.11 [48.65–81.11] ng/ml, *p* < 0.001). MPO significantly correlated with left atrium volume (LAV) and there existed a significant interaction between the two in relation to AF recurrence (*p* for interaction <0.05). During a median follow-up of 14 months, 28 patients with paroxysmal AF (12.44%) and 27 patients with persistent AF (25.47%) presented with recurrence after catheter ablation. The percentage of recurrence increased stepwise with increasing tertiles of MPO levels in both paroxysmal AF and persistent AF. MPO levels remained independently associated with AF recurrence after adjusting for potential confounding variables.

**Conclusion:**

MPO levels were higher in persistent AF than in paroxysmal AF and MPO was positively correlated with LAV in AF. Elevated MPO levels may predispose a switch in AF phenotype and AF recurrence after catheter ablation.

## 1. Introduction

Atrial fibrillation (AF) is the most common arrhythmic disorder, and its incidence continues to increase in an expanding global elderly population (1). It stands out as the major contributors to morbidity and mortality worldwide (2). AF progression mainly manifests as a transition from paroxysmal AF to persistent AF, the pathogenic mechanism of which is multifactorial and not fully understood, including electrical and anatomical atrial remodeling, and left atrial (LA) enlargement (3, 4). AF progression itself is associated with an increased risk of AF recurrence and other clinical adverse events, such as embolism or stroke (5). Although catheter ablation has emerged as the most common and important intervention strategy to control the rhythm and improve clinical symptoms, substantial patients experience post-ablation AF recurrence (6).

Myeloperoxidase (MPO), a neutrophil-derived heme enzyme, is gaining increased attention owing to its association with various cardiovascular diseases. Atrial fibrosis, combined with electrical remodeling and contractile dysfunction, has been recognized as the substrate of AF, and is a key factor in the progression from paroxysmal AF to persistent AF (7). MPO is considered a crucial profibrotic mediator for structural alterations in AF (8). Additionally, MPO acts as a leukocyte activation marker and its high abundance in leukocytes, which have been reported to accumulate in patients with AF, may play a prominent role in the initiation and maintenance of AF.

Recent studies have demonstrated that MPO levels are higher in patients with AF than in individuals without AF and are 10-fold higher in the LA than in the periphery (9). In patients with paroxysmal AF, elevated plasma MPO levels confer an increased risk of AF recurrence after catheter ablation (10). However, MPO levels in different clinical AF phenotypes remain unclear. This study aimed to explore whether plasma MPO levels vary in paroxysmal and persistent AF and their role in predicting AF recurrence after catheter ablation.

## 2. Materials and methods

This study was approved by the ethics committee of Ruijin Hospital, Shanghai Jiao Tong University School of Medicine, and conducted according to the Declaration of Helsinki. Written informed consent was obtained from all participants.

### 2.1. Patient population

**Figure 1:**
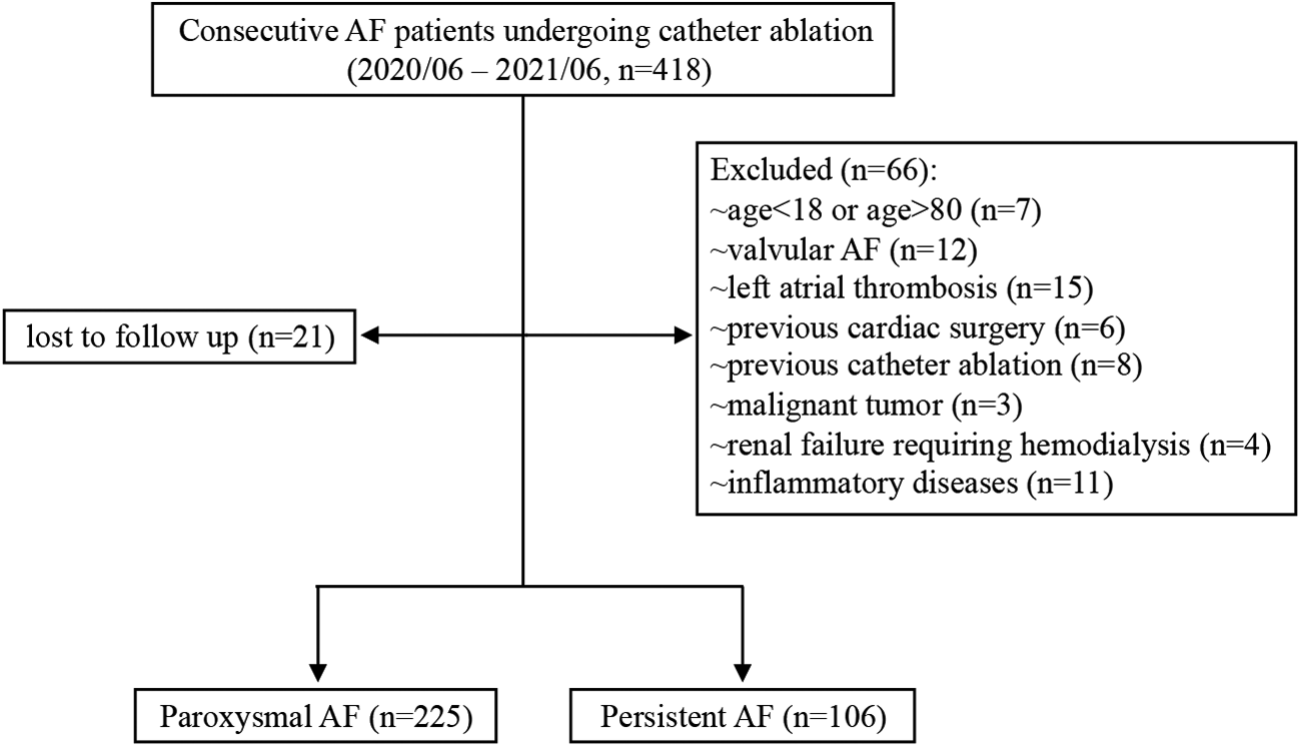
Flowchart of recruitment procedure. AF, atrial fibrillation.

A total of 331 consecutive AF patients undergoing first radiofrequency catheter ablation in Shanghai Ruijin Hospital between June 2020 and June 2021 were recruited in this study. The following exclusion criteria were applied to avoid confounding data: age <18 or >80 years (*n* = 7), valvular AF (*n* = 12), left atrial (LA)/LA appendage thrombus (*n* = 15), previous cardiac surgery (*n* = 6), previous catheter ablation (*n* = 8), malignant tumor (*n* = 3), renal failure requiring hemodialysis (*n* = 4) and inflammatory diseases (*n* = 11). Additionally, 21 patients were lost to follow-up. Diagnosis of paroxysmal or persistent AF was based on the latest guidelines (11). Transthoracic and transesophageal echocardiography were performed in all patients prior to ablation. Ultimately, 225 and 106 patients with paroxysmal and persistent AF were included in the final analysis, respectively (Figure 1).

### 2.2. Ablation procedure

All patients included in this study underwent catheter ablation using Robotic magnetic navigation (RMN) as previously described (12). For electroanatomic mapping and ablation, connecting an open-irrigated ablation catheter (NaviStar™ RMT Ther-moCool™; Biosense Webster, CA, USA) to a 3D mapping system (CARTO™, Biosense Webster, CA, USA) and the RMN Niobe™ ES system (Stereotaxis Inc., St. Louis, MO, USA). For patients with paroxysmal AF, additional fracture potential ablation or linear ablation was performed when necessary. The RF current was delivered for 30–40 s per lesion, applying 30–40 W (irrigation flow rate 17 ml/min) with a generator (Stockert, Biosense Webster, CA, USA) in a power-controlled mode. Power was selected based on the location of the catheter tip in the LA. For patients with persistent AF, electrical cardioversion was attempted once pulmonary vein isolation was achieved. For patients whose rhythm could not be converted to the sinus rhythm, the LA roof line lesion was created by the RMN catheter to ease subsequent electrical cardioversion. Substrate modifications, such as ablation of complex fractionated atrial electrogram, were not allowed in this study.

### 2.3. Biochemical assessments

Blood samples of all patients with AF were collected from the femoral vein peri-interventionally before ablation. The demographics and clinical characteristics of all participants were obtained from the electronic medical recording system by trained physicians blinded to the aim of the analysis. Serum levels of high-sensitivity C-reactive protein (hsCRP) were determined using enzyme-linked immunosorbent assay (Biocheck Laboratories, Toledo, OH, USA). Estimated glomerular filtration rate (eGFR) was calculated using the Chronic Kidney Disease Epidemiology Collaboration equation (13). For assessment of plasma MPO levels, blood samples were collected in anticoagulant-coated tubes, which were transferred at freezing temperatures and centrifuged within 30 min of collection at 3,000 rpm for 15 min to obtain plasma. All samples were stored at −80°C before analysis. Serum level of MPO was measured using colloidal gold immunochromatographic assay (GICA) (Eachy biopharma, China).The measurement range was 3–500 ng/ml, according to the manufacturer instructions. The average inter- and intra-assay coefficients of variability were 5.2%–4.6%, respectively.

### 2.4. Follow-up

All AF patients were followed up with a 12-lead electrocardiogram, 24 h Holter recordings, and telephone inquiries after catheter ablation. Recurrence was defined as the occurrence of confirmed atrial tachyarrhythmia which includes sustained AF (lasting >30 s) and atrial flutter or atrial tachycardia more than three months after the catheter ablation.

### 2.5. Statistical analysis

Statistical analyses were performed using SPSS 26.0 software (IBM, Armonk, New York, NY, USA). Continuous variables were expressed as mean ± standard deviation (SD) if data were normally distributed, median (25th-75th percentile) otherwise, and categorical variables were expressed as frequency (percentage). Differences between groups were compared using the *t*-test or Mann–Whitney *U* test for continuous variables, and for categorical variables, the between-group differences were assessed using the chi–square test. Spearman's correlation test was used to assess the relationship between the plasma MPO levels and LA volume (LAV). Univariate and multivariate Cox proportional hazards regression analyses were performed to examine the relationship between AF recurrence and plasma MPO levels. The MPO was included in the Cox proportional hazards regression model analysis as a log-transformed continuous variable, ordinal variable, or a categorical variable divided into tertiles. Receiver operating characteristic (ROC) curves were plotted to determine the ability of the MPO level to predict AF recurrence and the areas under the curve (AUC) were compared using the DeLong method. Statistical significance was set at two-sided *p* < 0.05.

## 3. Results

### 3.1. Clinical characteristics

This study finally included 225 patients with paroxysmal AF and 106 patients with persistent AF. Their baseline characteristics are shown in Table 1. There were no differences in age, sex, body mass index (BMI), or the presence of hypertension, diabetes, hyperlipidemia, or coronary heart disease between both AF groups. A higher percentage of patients with persistent AF were smokers. The left atrial diameter (LAD) and volume (LAV) were larger and left ventricular ejection fraction (LVEF) was lower in patients with persistent AF.

**Table 1:** Baseline characteristics of AF patients.

	paroxAF (*n* = 225)	persAF (*n* = 106)	*P V*alue
Male, *n* (%)	157 (69.3)	78 (73.6)	0.428
Age, year	63.96 ± 11.31	64.42 ± 10.12	0.724
Body mass index, kg/m^2^	24.71 ± 3.55	25.37 ± 3.19	0.103
Smoking, *n* (%)	49 (21.8)	43 (40.6)	<0.001
Hypertension, *n* (%)	127 (56.4)	64 (60.4)	0.499
Systolic blood pressure, mm Hg	136.01 ± 19.24	131.18 ± 19.64	0.035
Diastolic blood pressure, mm Hg	76.73 ± 11.70	81.44 ± 13.52	0.002
DM, *n* (%)	35 (15.6)	22 (20.8)	0.242
Dyslipidemia, *n* (%)	27 (12.0)	11 (10.4)	0.666
Stroke, *n* (%)	4 (1.8)	5 (4.7)	0.125
Coronary artery disease	50 (22.2)	31 (29.2)	0.166
Serum creatinine, µmol/L	79.26 ± 17.31	81.90 ± 15.64	0.183
eGFR, ml/min per 1.73 m^2^	84.70 (72.45–93.00)	82.70 (70.25–92.50)	0.449
hsCRP, mg/L	0.74 (0.37–1.74)	0.77 (0.43–1.98)	0.451
MPO, ng/ml	56.31 (40.33–73.51)	64.11 (48.65–81.11)	0.001
LAD, mm	39.58 ± 3.97	44.28 ± 4.18	<0.001
LVEF, %	66.12 ± 5.33	61.07 ± 9.29	<0.001
LAV, cm^3^	118.76 ± 26.19	141.42 ± 29.99	<0.001
CHA2DS2-VASc score	2.05 ± 1.45	2.30 ± 1.58	0.151

AF, atrial fibrillation; paroxAF, paroxysmal atrial fibrillation; persAF, persistent atrial fibrillation; DM, diabetes mellitus; eGFR, estimated glomerular filtration rate; hsCRP, high-sensitivity C reactive protein; MPO, myeloperoxidase; LAD, left atrial diameter; LVEF, left ventricular ejection fraction; LAV, left atrial volume.

### 3.2. MPO levels with AF progression

**Figure 2:**
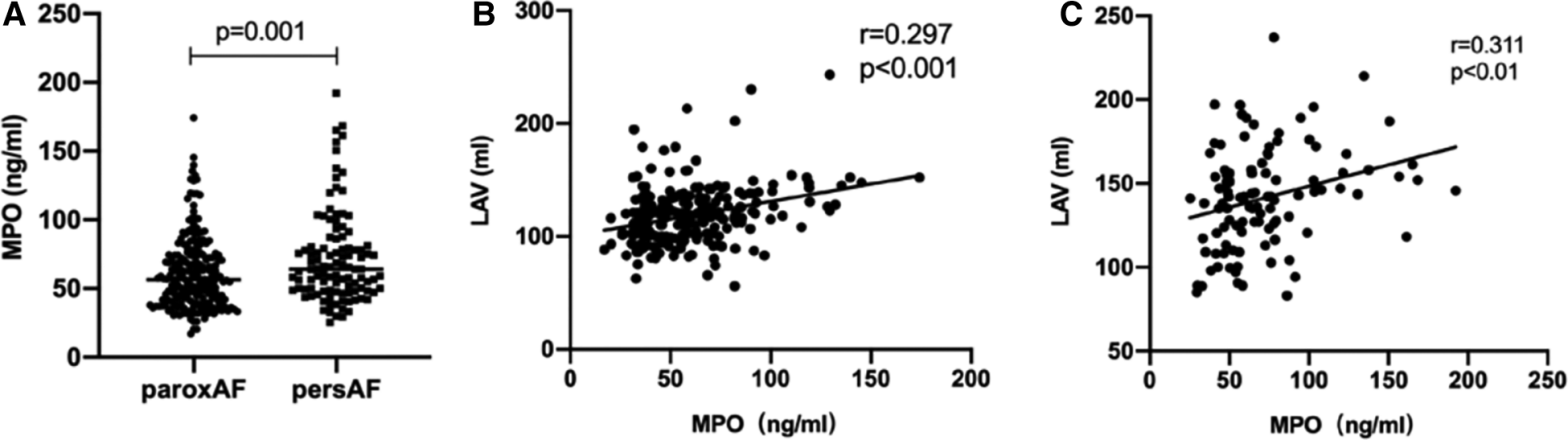
Relationship between MPO and AF progression. Comparison of plasma MPO levels between patients with paroxysmal AF and persistent AF, *n* = 225 106 for paroxysmal AF; *n* = 106 for persistent AF, *p* = 0.001 (**A**) Spearman correlation coefficient analysis between MPO and LAV in patients with paroxysmal AF, *n* = 225, *r* = 0.297, *p* < 0.001 (**B**) and persistent AF, *n* = 106, *r* = 0.311, *p* < 0.01 (**C**) MPO: myeloperoxidase; AF, atrial fibrillation; LAV, left atrial volume.

Plasma MPO levels were higher in persistent AF than in paroxysmal AF (64.11 [48.65–81.11] vs. 56.31 [40.33–73.51] ng/ml, *p* = 0.001). Furthermore, a significant positive correlation was observed between plasma MPO levels and LAV in both AF groups (paroxysmal AF: *r* = 0.297, *p* < 0.001; persistent AF: *r* = 0.311, *p* < 0.001; Figure 2). Despite adjusting for confounding factors, plasma MPO levels positively correlated with LAV in both paroxysmal and persistent AF groups (Table 2).

**Table 2:** Correlation between MPO and LAV.

	Unadjusted *r*	Unadjusted *p*	*Adjusted *r*	*Adjusted *p*
paroxAF	0.297	<0.001	0.334	<0.001
persAF	0.311	0.001	0.267	0.006

* Adjusted for age, sex, body mass index, smoking, hypertension, diabetes, dyslipidemia, stroke, coronary artery disease, eGFR, LAD, LVEF for paroxAF; adjusted for age, sex, body mass index, smoking, hypertension, diabetes, dyslipidemia, stroke, coronary artery disease, eGFR, LAD, LVEF, duration of AF for persAF. paroxAF, paroxysmal atrial fibrillation; persAF, persistent atrial fibrillation.

### 3.3. MPO levels with AF recurrence after catheter ablation

**Figure 3:**
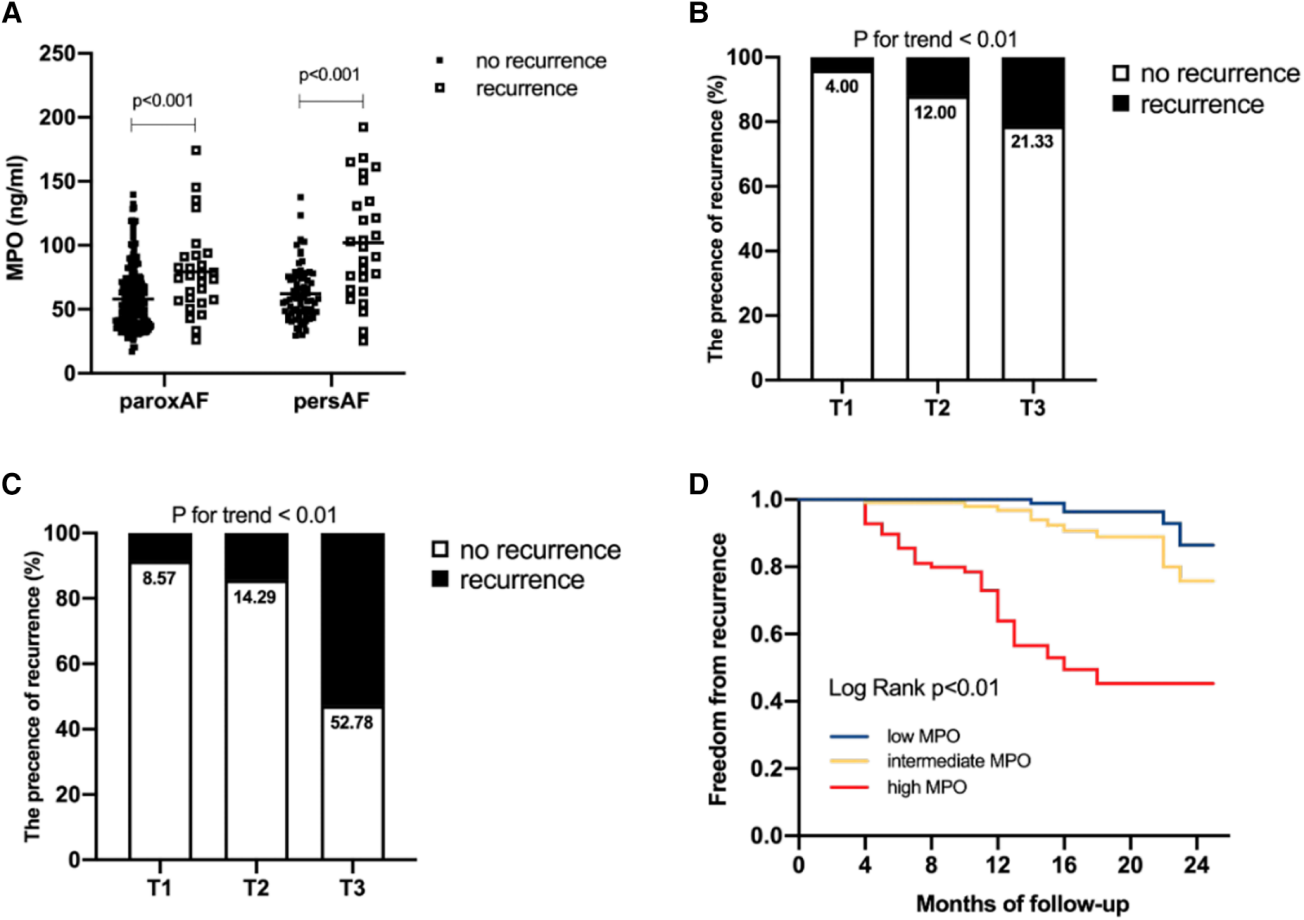
Association of plasma MPO levels with AF recurrence after catheter ablation. Comparison of plasma MPO levels between recurrence group and non-recurrence group in paroxysmal AF and persistent AF (**A**) AF recurrence across the tertiles of plasma MPO in paroxysmal AF, *p* for trend <0.01 (**B**) and persistent AF, *p* for trend <0.01 (**C**) Kaplan–Meier curves demonstrated recurrence of AF across tertiles of MPO, *p* < 0.01 (**D**).

**Figure 4:**
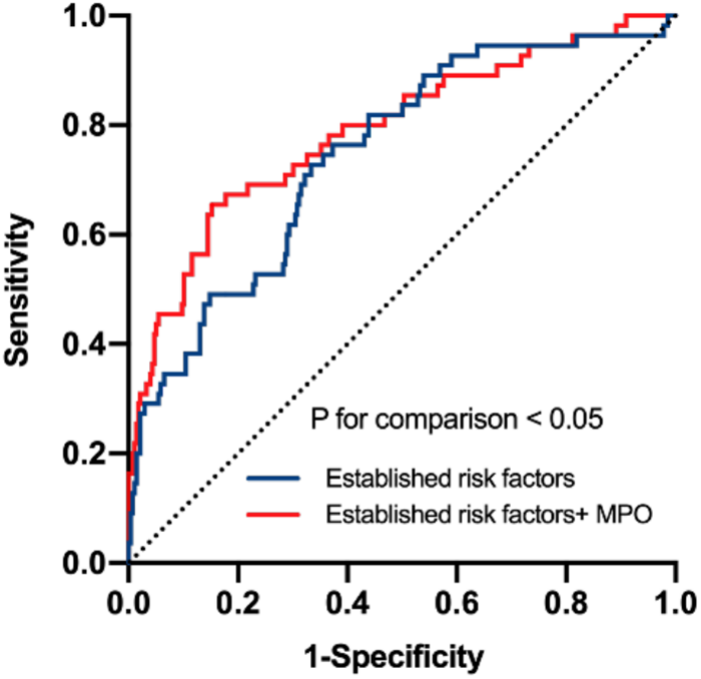
Receiver operating characteristic curve analysis to verify the predictive value of MPO. Established risk factors: age, sex, body mass index, smoking, hypertension, diabetes, dyslipidemia, stroke, coronary artery disease, eGFR, LAD, LVEF, LAV, duration of AF. *p* for comparison <0.05.

**Figure 5:**
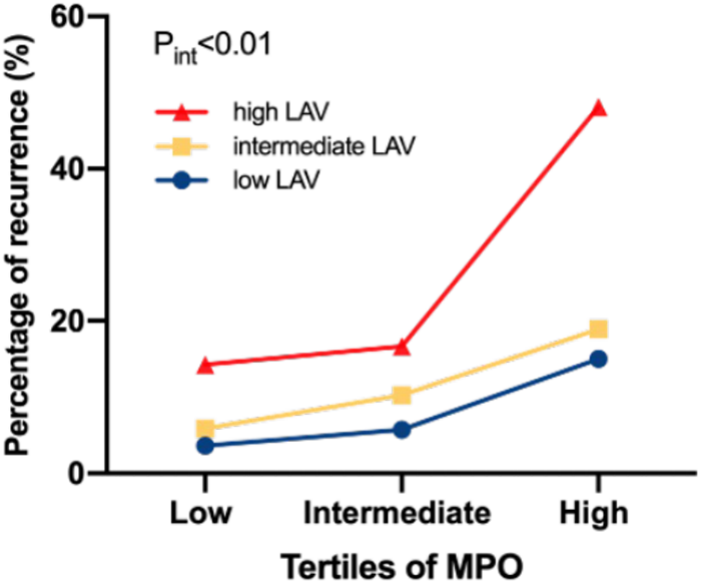
Percentage of AF recurrence in relation to interactions between plasma MPO and LAV. *p* for interaction <0.01.

In this study, 28 of 225 (12.44%) patients with paroxysmal AF and 27 of 106 (25.47%) patients with persistent AF experienced AF recurrence after catheter ablation, within a median follow-up of 14 months. As shown in Figure 3, plasma MPO levels were higher in recurrence group than in non-recurrence either in paroxysmal or persistent AF. We observed stepwise increases in the presence of AF recurrence from the lowest tertile to the highest plasma MPO in both AF groups (*p* for all trends <0.01; Figure 3). There was a statistically significant difference in AF recurrence-free survival rates from the lowest to the highest tertile of MPO (log-rank test, *p* < 0.01; Figure 3). Cox analysis confirmed that MPO level was an independent risk factor for post-catheter ablation AF recurrence after adjustment for age, sex, BMI, smoking, hypertension, diabetes, dyslipidemia, stroke, coronary artery disease, eGFR, LAD, LVEF, LAV, and duration of AF, regardless of MPO as a continuous variable, a categorical variable or an ordinal variable (Table 3). As depicted in the ROC curve, the inclusion of plasma MPO level in the established risk factors significantly improved the diagnostic performance for AF recurrence (AUC: 0.748 [0.701–0.794] vs. 0.705 [0.655–0.755], *p* < 0.05; Figure 4). Furthermore, when patients were classified according to tertiles of MPO and LAV, there was interaction between plasma MPO levels and LAV on the recurrence of AF after catheter ablation (*p* for interaction <0.01; Figure 5).

**Table 3:** Uni- and multi-variant cox analysis.

	Unadjusted HR	*p*-value	Adjusted for Model 1 HR	*p*-value	Adjusted for Model 2 HR	*p*-value
Log_10_ MPO per SD	3.001 (2.256–3.991)	<0.001	3.002 (2.247–4.010)	<0.001	3.013 (2.145–4.232)	<0.001
MPO tertiles	3.345 (2.240–4.995)	<0.001	3.439 (2.291–5.162)	<0.001	3.357 (2.121–5.315)	<0.001
1st	Ref		Ref		Ref	
2st	1.972 (0.776–5.012)	0.154	1.879 (0.737–4.792)	0.186	1.705 (0.662–4.392)	0.269

Model 1: adjusted for age, sex, body mass index.

Model 2: adjusted for age, sex, body mass index, smoking, hypertension, diabetes, dyslipidemia, stroke, coronary artery disease, eGFR, LAD, LVEF, LAV, duration of AF.

## 4. Discussion

### 4.1. Main findings

This study is the first to report that plasma MPO levels were higher in patients with persistent AF than in those with paroxysmal AF; circulating MPO levels were associated with AF progression. We also demonstrated, for the first time, a significant correlation between plasma MPO levels and LAV. Plasma MPO levels were associated with recurrence after catheter ablation, independent of traditional risk factors.

### 4.2. MPO with AF progression and adverse outcomes after catheter ablation

AF progression, defined as the transition from paroxysmal to persistent AF, is clinically important and is closely related to the management of patients with AF. Although not all cases of paroxysmal AF progress to persistent AF, one Canadian Registry of Atrial Fibrillation followed patients with paroxysmal AF for 10 years and found that more than half of these patients developed persistent AF or died (14). Risk factors for AF progression are similar to those for AF occurrence. We found that plasma MPO levels were higher in patients with persistent AF than those with paroxysmal AF. Furthermore, plasma MPO levels were positively correlated with LAV in paroxysmal AF and persistent AF. Excessive MPO release reportedly exacerbates oxidative stress, leading to various metabolic disorders and cardiovascular diseases, such as heart failure and myocardial ischemia (15). Leukocyte-derived heme enzyme MPO could generates hypochlorous acid (HOCl) (16, 17). It is an important regulatory switch of matrix metalloproteinase activity, which is closely related to extracellular matrix remodeling in AF (8). Accumulating evidence have demonstrated that AF exhibits pathophysiological characteristics of inflammatory disease, and atrial fibrosis manifested by ECM turnover stands exactly at the intersection of inflammation and AF (18). In our cohort, higher plasma MPO levels in persistent AF than in paroxysmal AF may indicate that persistent AF presents with more pronounced remodeling and is more refractory. Additionally, increased LAV is a known risk factor for AF occurrence and recurrence (19). Recently, several reports have found that LAV is strongly associated with AF progression (14, 20–22). An enlarged LAV is a hallmark of atrial remodeling through many mechanisms, including pressure overload, ageing, oxidative stress, and inflammation. The positive correlation between plasma MPO levels and LAV indicates that they may share some of the crucial mechanisms in progressive remodeling. A previous study reported no statistical difference in the circulating MPO levels between different AF phenotypes, which is in contrast to our results, probably due to their small sample size (*n* = 55 for paroxysmal AF; *n* = 58 for persistent AF) (9). Additionally, we found that patients with AF recurrence after catheter ablation had higher plasma MPO levels than those without recurrence. The percentage of AF recurrence increased stepwise from the lowest to the highest tertiles of plasma MPO levels in both paroxysmal AF and persistent AF. Moreover, AF recurrence-free survival rates significantly varied across the MPO tertiles. After adjusting for traditional risk factors, MPO still presented a notable value for AF recurrence after catheter ablation, which was consistent with the findings of Li et al. (10). In this study, an interaction was observed between plasma MPO levels and LAV in relation to AF recurrence after catheter ablation, indicating that higher circulating MPO levels may predispose to more significant atrial remodeling characterized by LA dilation. Adding MPO into the traditional risk factors that include LAV significantly increased the predictive performance for AF recurrence, suggesting the possibility of unexplored mechanisms behind the role of MPO in adverse outcomes after catheter ablation, besides the common factors shared with LAV enlargement.

### 4.3. Clinical implications

The increasing risk of adverse cardiovascular events during the natural progressive course of AF warrants the early identification of possible risk factors for AF progression and treatment of underlying conditions. Plasma MPO levels are closely associated with the progressive course and are easily detectable as peripheral markers. Although AF is a multifaceted arrhythmic disorder, plasma MPO levels may help better stratify AF patients along with LA imaging and clinical characteristics. In our study, plasma MPO level was an independent risk factor for AF recurrence, which may contribute to establishing more optimal patient selection for catheter ablation and development of a more individualized treatment strategy.

### 4.4. Limitations

This study had some limitations owing to its retrospective, cross-sectional nature. We observed associations, but could not establish a causal relationship. Due to differences in the time of patient enrollment, the follow-up durations were not wholly consistent. Although basic clinical features were consistently distributed in the paroxysmal and persistent AF groups, selection bias and unknown confounding factors that may have affected circulating MPO levels could not be excluded.

## 5. Conclusions

This study demonstrates that plasma MPO levels are higher in persistent AF than in paroxysmal AF and are thus associated with AF progression. Plasma MPO levels are positively correlated with LAV in AF patients. Elevated plasma MPO levels grant an increased risk of AF recurrence after catheter ablation. This study provides new information on the pathophysiology of AF progression and adverse outcomes post catheter ablation.

## Data Availability

The original contributions presented in the study are included in the article/Supplementary Material, further inquiries can be directed to the corresponding authors.

## References

[1] DeLago AJ Essa M Ghajar A Hammond-Haley M Parvez A Nawaz I Incidence and mortality trends of atrial fibrillation/atrial flutter in the United States 1990 to 2017. Am J Cardiol. (2021) 148:78–83. 10.1016/j.amjcard.2021.02.014 33640365

[2] Lloyd-Jones DM Wang TJ Leip EP Larson MG Levy D Vasan RS Lifetime risk for development of atrial fibrillation: the framingham heart study. Circulation. (2004) 110:1042–6. 10.1161/01.CIR.0000140263.20897.42 15313941

[3] Gunawardene MA Willems S. Atrial fibrillation progression and the importance of early treatment for improving clinical outcomes. Europace. (2022) 24:ii22–28. 10.1093/europace/euab257 35661866

[4] Jalife J Kaur K. Atrial remodeling, fibrosis, and atrial fibrillation. Trends Cardiovasc Med. (2015) 25:475–84. 10.1016/j.tcm.2014.12.015 25661032 PMC5658790

[5] Potpara TS Stankovic GR Beleslin BD Polovina MM Marinkovic JM Ostojic MC A 12-year follow-up study of patients with newly diagnosed lone atrial fibrillation: implications of arrhythmia progression on prognosis: the Belgrade atrial fibrillation study. Chest. (2012) 141:339–47. 10.1378/chest.11-0340 21622553

[6] Du H Yang L Hu Z Zhang H. Anxiety is associated with higher recurrence of atrial fibrillation after catheter ablation: a meta-analysis. Clin Cardiol. (2022) 45:243–50. 10.1002/clc.23753 35043425 PMC8922539

[7] Friedrichs K Baldus S Klinke A. Fibrosis in atrial fibrillation—role of reactive species and MPO. Front Physiol. (2012) 3:214. 10.3389/fphys.2012.00214 22723783 PMC3379725

[8] Rudolph V Andrie RP Rudolph TK Friedrichs K Klinke A Hirsch-Hoffmann B Myeloperoxidase acts as a profibrotic mediator of atrial fibrillation. Nat Med. (2010) 16:470–4. 10.1038/nm.2124 20305660 PMC2880896

[9] Holzwirth E Kornej J Erbs S Obradovic D Bollmann A Hindricks G Myeloperoxidase in atrial fibrillation: association with progression, origin and influence of renin-angiotensin system antagonists. Clin Res Cardiol. (2020) 109:324–30. 10.1007/s00392-019-01512-z 31236695

[10] Li SB Yang F Jing L Ma J Jia YD Dong SY Myeloperoxidase and risk of recurrence of atrial fibrillation after catheter ablation. J Investig Med. (2013) 61:722–7. 10.2310/JIM.0b013e3182857fa0 23392057

[11] Hindricks G Potpara T Dagres N Arbelo E Bax JJ Blomstrom-Lundqvist C 2020 ESC guidelines for the diagnosis and management of atrial fibrillation developed in collaboration with the European association for cardio-thoracic surgery (EACTS): the task force for the diagnosis and management of atrial fibrillation of the European society of cardiology (ESC) developed with the special contribution of the European heart rhythm association (EHRA) of the ESC. Eur Heart J. (2021) 42:373–498. 10.1093/eurheartj/ehaa612 32860505

[12] Li X Jin Q Zhang N Ling T Lin C Jia K Procedural outcomes and learning curve of cardiac arrhythmias catheter ablation using remote magnetic navigation: experience from a large-scale single-center study. Clin Cardiol. (2020) 43:968–75. 10.1002/clc.23391 32453461 PMC7462195

[13] Levey AS Stevens LA Schmid CH Zhang YL Castro AF 3rd Feldman HI A new equation to estimate glomerular filtration rate. Ann Intern Med. (2009) 150:604–12. 10.7326/0003-4819-150-9-200905050-00006 19414839 PMC2763564

[14] Padfield GJ Steinberg C Swampillai J Qian H Connolly SJ Dorian P Progression of paroxysmal to persistent atrial fibrillation: 10-year follow-up in the Canadian registry of atrial fibrillation. Heart Rhythm. (2017) 14:801–7. 10.1016/j.hrthm.2017.01.038 28232263

[15] Ndrepepa G. Myeloperoxidase—a bridge linking inflammation and oxidative stress with cardiovascular disease. Clin Chim Acta. (2019) 493:36–51. 10.1016/j.cca.2019.02.022 30797769

[16] Fu X Kassim SY Parks WC Heinecke JW. Hypochlorous acid oxygenates the cysteine switch domain of pro-matrilysin (MMP-7). A mechanism for matrix metalloproteinase activation and atherosclerotic plaque rupture by myeloperoxidase. J Biol Chem. (2001) 276:41279–87. 10.1074/jbc.M106958200 11533038

[17] Fu X Kassim SY Parks WC Heinecke JW. Hypochlorous acid generated by myeloperoxidase modifies adjacent tryptophan and glycine residues in the catalytic domain of matrix metalloproteinase-7 (matrilysin): an oxidative mechanism for restraining proteolytic activity during inflammation. J Biol Chem. (2003) 278:28403–9. 10.1074/jbc.M304739200 12759346

[18] Issac TT Dokainish H Lakkis NM. Role of inflammation in initiation and perpetuation of atrial fibrillation: a systematic review of the published data. J Am Coll Cardiol. (2007) 50:2021–8. 10.1016/j.jacc.2007.06.054 18021867

[19] Zhuang J Wang Y Tang K Li X Peng W Liang C Association between left atrial size and atrial fibrillation recurrence after single circumferential pulmonary vein isolation: a systematic review and meta-analysis of observational studies. Europace. (2012) 14:638–45. 10.1093/europace/eur364 22117033

[20] De With RR Marcos EG Dudink E Spronk HM Crijns H Rienstra M Atrial fibrillation progression risk factors and associated cardiovascular outcome in well-phenotyped patients: data from the AF-RISK study. Europace. (2020) 22:352–60. 10.1093/europace/euz339 31865391

[21] Malavasi VL Fantecchi E Tordoni V Melara L Barbieri A Vitolo M Atrial fibrillation pattern and factors affecting the progression to permanent atrial fibrillation. Intern Emerg Med. (2021) 16:1131–40. 10.1007/s11739-020-02551-5 33161524

[22] Csecs I Yamaguchi T Kheirkhahan M Czimbalmos C Fochler F Kholmovski EG Left atrial functional and structural changes associated with ablation of atrial fibrillation—cardiac magnetic resonance study. Int J Cardiol. (2020) 305:154–60. 10.1016/j.ijcard.2019.12.010 31874788

